# Effect of single tube sections on the structural safety of Chinese solar greenhouse skeletons

**DOI:** 10.1038/s41598-021-98779-y

**Published:** 2021-09-29

**Authors:** Xingan Liu, Zhenkun Li, Lei Zhang, Yu Liu, Yiming Li, Tianlai Li

**Affiliations:** 1 College of Horticulture, Shenyang Agricultural University, Shenyang, 110866 China; 2 National & Local Joint Engineering Research Center of Northern Horticultural Facilities Design & Application Technology (Liaoning), Shenyang, 110866 China; 3 College of Engineering, Shenyang Agricultural University, Shenyang, 110866 China; 4 College of Information and Electrical Engineering, Shenyang Agricultural University, Shenyang, 110866 China

**Keywords:** Plant sciences, Energy science and technology, Engineering

## Abstract

In recent years, the use of single-tube skeletons for the construction of Chinese solar greenhouses has increased. As a consequence, during the selection of the construction materials, the safety of these structures has become an important issue. The single tube section has various forms, but there is no scientific theory to guide the selection process. To the best of our knowledge, the scientific analysis of the impact of single pipe cross section on the safety of greenhouse skeleton has not been addressed so far. In this context, the finite element analysis software was used to calculate and analyze the stress elements, displacement of round tube, Ω tube, elliptic tube and square tube under the same load conditions. We used the Chinese Standard values as a reference and analyzed structural features of different sizes and thicknesses of the greenhouse steel skeleton sections under non-uniform snow load. The results showed that, under the same load condition, the maximum stress in the four skeleton materials was all located at the connection of the transverse tension bar and the front roof. In addition, under same load condition, the greenhouse skeleton with elliptic tube presented the smallest cross-sectional displacement between the different materials tested. The effect of increasing the size of the greenhouse frame was better than that of increasing the greenhouse material thickness. All this work will provide theoretical guidance to the material selection of this structure.

## Introduction

Chinese solar greenhouses are agricultural facilities that have been used in north China for crop cultivation without additional heating during severe cold weather^1^. By the end of 2017, the total area of horticultural facilities in China had reached 3.7 million hm^2^^2^. A significant amount of research had been performed in order to improve overall size^3^, wall structure^4^, surface structure^5^ and skeleton structure^6,7^, among others. In recent years, a new structure called single pipe skeleton has been used to replace the truss structure in the solar greenhouse. However, single pipe skeleton parts provide less safety as compared with traditional trusses. In fact, it has been reported that several single pipe Chinese solar greenhouses that have been built or are under construction, especially those with the greenhouse skeleton structure, display many potential safety hazards^8^. Some of them have resulted in the collapse of greenhouses' structures and in consequence, great economic losses^9,10^. The lack of scientific data that can be used to determine construction parameters is the main cause of these accidents. In addition, in order to reduce economic costs, relatively low safety coefficients have been considered when calculating the geometrical parameters of single tube sections. Moreover, the selection of many geometrical parameters of single tube sections have been based on empirical estimations, which results in weak structures that cannot support various loads. Therefore, it is essential to investigate the effect of geometric parameters of single tube sections on the safety factors of Chinese solar greenhouse skeleton structures.

Several researchers have studied the mechanical behavior of greenhouse structures using experimental and numerical methods. They have investigated the truss sections of the glasshouse skeleton structures^11^, the stress on the struts and the whole structure^12^, and the influence of different structural parameters on the greenhouse characteristics^13^. These studies have provided the theoretical basis to improve safety and reduce economic costs of the solar greenhouse skeletons. Also, several researchers have studied the characteristics of single-tube skeletons in order to replace truss structures. Lu and Qiu carried out a load test of a single-slope solar greenhouse skeleton structure. For this specific study, they determined the influence of the truss structure, round-shaped steel tube (hereinafter referred to as round tube) structure, and square-shaped steel tube (hereinafter referred to as square tube) structure on the final capacity^14^. Also, a new type of full-steel frame with elliptic-shaped steel tube (hereinafter referred to as elliptic tube) was designed and tested for its safety^15^. Zhou included an elliptic pipe, a circular pipe, and Ω-shaped steel tube (hereinafter referred to as Ω tube) sections on their Chinese solar greenhouse design^16^. Even when a significant amount of research has been performed on single tube skeleton Chinese solar greenhouses, the influence of geometric parameters on structural safety still needs further study.

Compared with the truss structure, the single pipe skeleton does not consider various structural safety components. Thus, its safety is reduced. Within this context, the purpose of the present study was to perform a finite element analysis using several skeleton sections commonly used in greenhouses. The analysis, which considered different section dimensions and wall thicknesses, was performed to identify the best parameters. In addition, we calculated the single tube section of each span greenhouse. The results of the present investigation will provide theoretical support for the selection of proper parameters for the skeleton during the greenhouse construction.

## Methods

### Greenhouse structural parameters

**Figure 1:**
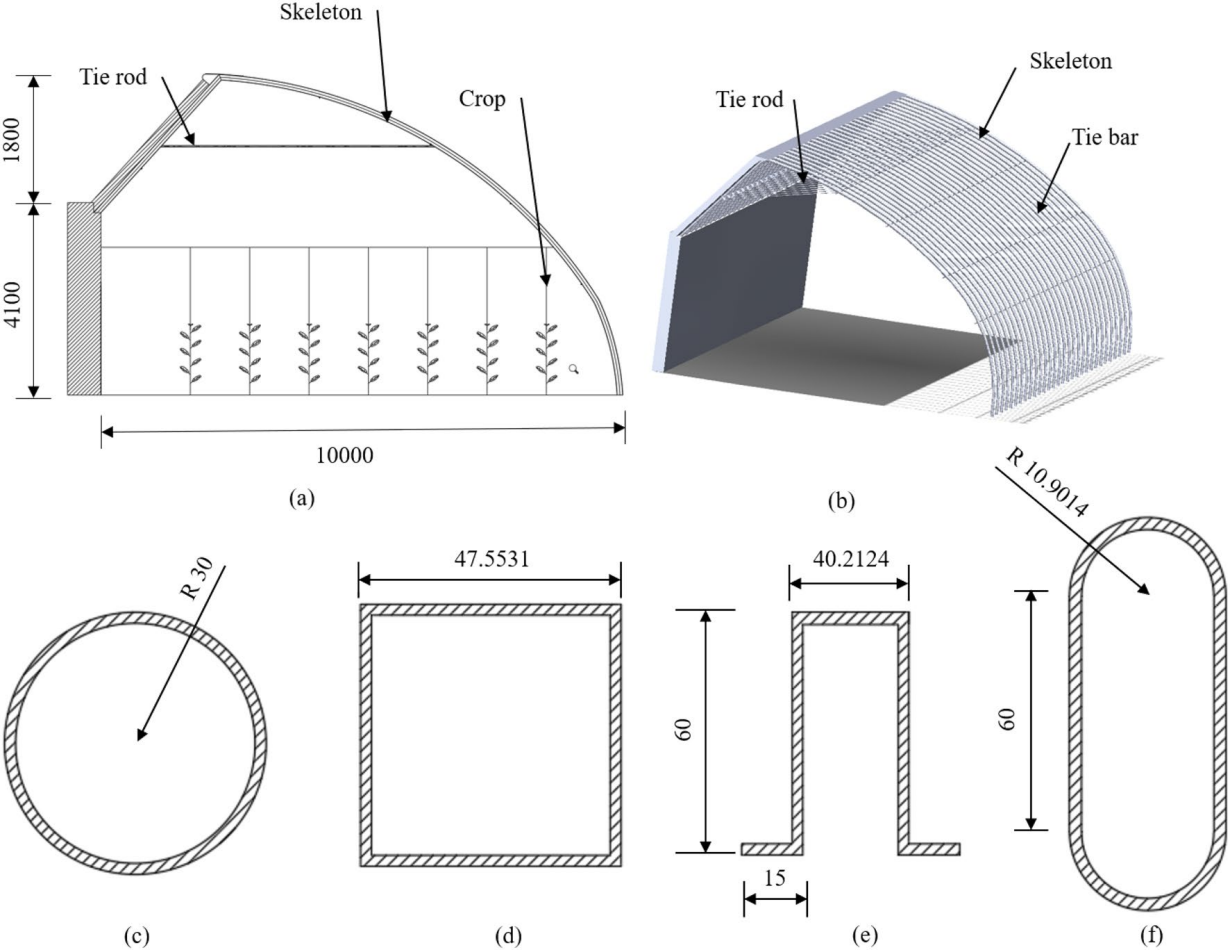
Explanation diagram of greenhouse skeleton structure (mm): (**a**) greenhouse sectional view; (**b**) greenhouse skeleton connection diagram; (**c**) round tube section size; (**d**) square tube section size; (**e**) Ω tube size; (**f**) elliptic tube section size.

The third-generation Liaoshen solar greenhouse, which is widely used in the Liaoning Province, has a high rate of land utilization. This solar greenhouse is selected in the present research. Greenhouse span is 10 m, ridge height is 6.1 m, greenhouse frame spacing is 0.85 m, and the horizontal plane of the north roof is 2.1 m. The front roof is an arched round roof with an arc length of about 12.3 m. The lower part of the north roof is covered with wood boards, the middle layer is made of benzene boards, and the outer layer included waterproof coiled materials. In addition, the tie rod and tie bar reinforcement are made of a 20 × 2 mm round tube. The rest of the parameters are shown in Fig. 1.

### Pipe shape and size

In this research, we determined the effect of the pipe shape and size on the greenhouse skeleton characteristics. The area of the round, elliptic, square and Ω tube sections were equal. Also, the wall thickness of the round, elliptic, square and Ω tube sections were 2 mm and the Q235 steel was used to build the greenhouse skeleton. Parameters of the Q235 steel used in the present research are shown in Table 1 and section sizes are shown in Fig. 1.

**Table 1:** Q235 steel parameters.

Category	Density/kg m^−3^	Elasticity modulus/GPa	Poisson ratio	Yield strength/MPa
Q235	7850	206	0.3	235

In the case of the elliptic tube, we also used the third-generation Liaoshen solar greenhouse structure steel arch. The tie rod was 20 × 2 mm. The tie bar was 20 × 2 mm. Tables 2 and 3 display the different main beam sizes and main beam thickness that were selected in the present study to determine their effect on the safety features of the greenhouse skeleton.

**Table 2:** Characteristics of the greenhouse frame materials.

Serial number	1	2	3	4	5	6
Main beam size/mm	25 × 40 × 2	25 × 45 × 2	25 × 50 × 2	30 × 60 × 2	30 × 65 × 2	30 × 70 × 2
Serial number	7	8	9	10	11	12
Main beam size/mm	30 × 75 × 2	30 × 80 × 2	30 × 85 × 2	30 × 90 × 2	30 × 95 × 2	30 × 100 × 2

**Table 3:** Wall thickness of main beam materials.

Serial number	1	2	3	4	5
Main beam material wall thickness/mm	30 × 60 × 1	30 × 60 × 1.5	30 × 60 × 2	30 × 60 × 2.5	30 × 60 × 3

### Geometric modeling and meshing

The structural performances are investigated by finite element method based on the ANSYS 18.0 software. In this research, we have employed the equilibrium equation, the geometric equation and the physical equation. The formula is as follows:

The equilibrium equation is expressed as:

1 $$\frac{{\partial \sigma_{{\text{x}}} }}{{\partial_{x} }} + \frac{{\partial \tau_{yx} }}{{\partial_{y} }} + \frac{{\partial \tau_{zx} }}{{\partial_{z} }} + F_{x} = 0$$

2 $$\frac{{\partial \sigma_{y} }}{{\partial_{y} }} + \frac{{\partial \tau_{yx} }}{{\partial_{x} }} + \frac{{\partial \tau_{zy} }}{{\partial_{z} }} + F_{y} = 0$$

3 $$\frac{{\partial \sigma_{z} }}{{\partial_{z} }} + \frac{{\partial \tau_{zx} }}{{\partial_{x} }} + \frac{{\partial \tau_{zy} }}{{\partial_{y} }} + F_{y} = 0$$

The stress state of an elastomer at any point in the body under the action of load can be composed of six stress components (i.e. σ_x_, σ_y_, σ_z_, τ_xy_, τ_yz_, τ_zx_).

The geometric equation is expressed as:

4 $$\varepsilon_{x} = \frac{{\partial_{u} }}{{\partial_{x} }},\gamma_{y} = \frac{{\partial_{w} }}{{\partial_{y} }} + \frac{{\partial_{v} }}{{\partial_{z} }}$$

5 $$\varepsilon_{y} = \frac{{\partial_{v} }}{{\partial_{y} }},\gamma_{y} = \frac{{\partial_{u} }}{{\partial_{z} }} + \frac{{\partial_{w} }}{{\partial_{x} }}$$

6 $$\varepsilon_{z} = \frac{{\partial_{w} }}{{\partial_{z} }},\gamma_{y} = \frac{{\partial_{v} }}{{\partial_{x} }} + \frac{{\partial_{u} }}{{\partial_{y} }}$$

The strain at any point in the elastic body can be represented by six components: γ_xy_, γ_yz_, γ_zx_ are all shear strains, and ε_x_, ε_y_, ε_z_ are all positive strains.

The physical equation is expressed as:

7 $$\sigma_{x} = \frac{E}{1 + v}\left( {\frac{v}{1 - 2v}\theta + \varepsilon_{x} } \right),\;\;\tau_{yz} = \frac{E}{2(1 + v)}\gamma_{yz}$$

8 $$\sigma_{y} = \frac{E}{1 + v}\left( {\frac{v}{1 - 2v}\theta + \varepsilon_{y} } \right),\;\;\tau_{zx} = \frac{E}{2(1 + v)}\gamma_{zx}$$

9 $$\sigma_{z} = \frac{E}{1 + v}\left( {\frac{v}{1 - 2v}\theta + \varepsilon_{z} } \right),\;\;\tau_{xy} = \frac{E}{2(1 + v)}\gamma_{xy}$$

The displacement of any point in an elastic body can be expressed by three displacement components (i.e. w, v and u) along the direction of the rectangular coordinate axis. E is the elastic modulus. ν is Poisson’s ratio.

We used the finite element software to study the stress and deformation distribution of the greenhouse skeleton structure under different load conditions can be directly understood^17^. This software can be used to accurately calculate different structural features and determine the deformation stress and strain of the structure. For this reason, the finite element software is an important tool used in the design of greenhouse structure^18^. Depending on structural characteristics of the greenhouse, the finite element model can be partly simplified considering the calculation efficiency. Thus, it was not necessary to establish a complete structural model. The load distribution was consistent throughout the length and only varied in the span-direction. Thus, analyzing a bay was equivalent to analyzing the entire structure. For this reason, a bay located in the middle of the whole skeleton was been selected for the analysis^19^. In the finite element model, some greenhouse parts that had small impact on the analysis were ignored. These included welding gaskets and vents. Although the greenhouse skeleton structure included curve sections, the single pipe skeleton might be considered as a series of straight lines. Considering this, we used the finite element software is used to analyze the mechanical properties of 4 tubular greenhouses.

### Unit and meshing

**Figure 2:**
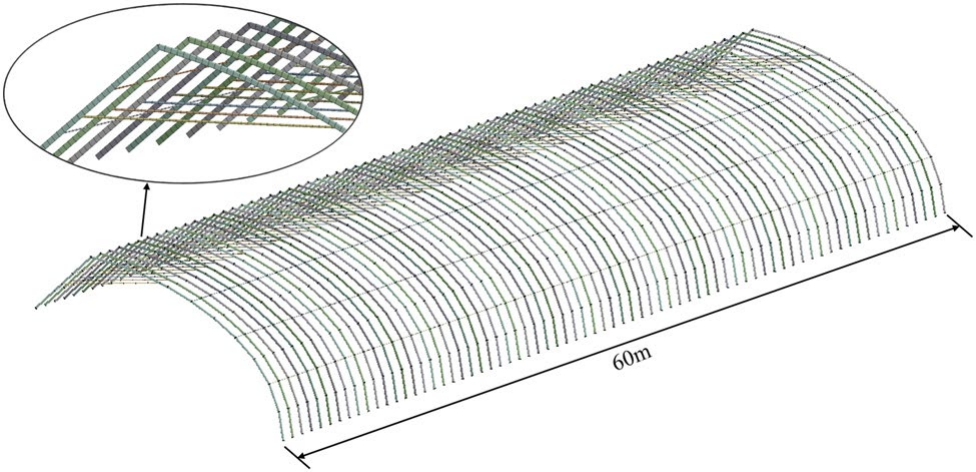
Schematic diagram of the greenhouse skeleton.

The Beam 188 is a 3-D 2-node beam element based on the Timoshenko beam theory which includes shear-deformation effects, and is suitable for analyzing slender to moderately stubby/thick beam structure. Since Beam 188 is suitable for the analysis of thin or thick beams, it is used as the grid division unit during the modeling of the greenhouse skeleton. According to the size of the greenhouse, 3-D modeling was carried out using the finite element software. In addition, during grid division in the solid modeling process, factors that did not affect strength calculation, such as chamfered walls, were ignored^20^. The finite element modeling was performed considering the greenhouse structural parameters, as shown in Fig. 2.

**Figure 3:**
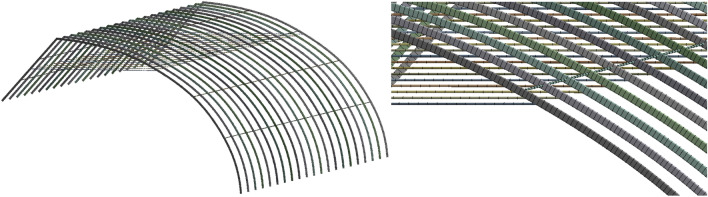
The finite element model and its details of the skeleton model.

It is worth mentioning that the web members are mainly subjected to only axial force in theory. Thus, the link element seems to be more suitable. The grid sizes of tie rods and tie bars were both controlled at 100 mm. And the grids were properly encrypted at connection of members to improve calculation accuracy. The finite element model and its details are displayed in Fig. 3.

### Constraint conditions

The tie bars are fixed on the skeleton by buckles, which can be regarded as rigid constraints. Therefore, the connection is made through sharing nodes. Meanwhile, the two ends of the skeleton were fixed on the base.

### Loads and analytical method

**Figure 4:**
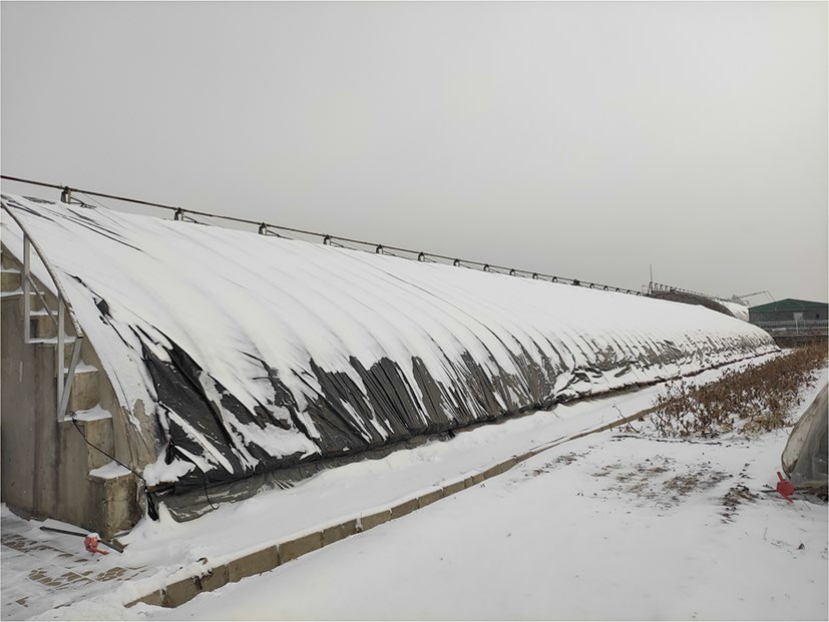
Field picture of greenhouse snow load.

The failure mode of the single-pipe solar greenhouse structure is the collapse of the greenhouse structure. In addition to the self-weight of the greenhouse structure, crop load and the concentrated load of the roof structure, the main external factor that causes the collapse of the greenhouse structure in Shenyang is the snow load. This snow load is shown in Fig. 4.

The external conditions of the greenhouse represent an important constrain. They include constant load (G), crop load (C_k_), concentrated load of roof construction (E_k_), seismic load (A_k_), and snow load (S_k_).

### Constant load G

The total value of the constant load on the greenhouse skeleton structure is G, which includes the skeleton weight G_0_, the quality of the front roof covering material G_1_, and the quality of the north roof material G_2_.

### Crop load C_k_

Crop load is the load formed by crop hanging on the greenhouse due to the need of cultivation, and its size is related to the variety of the cultivated crop. According to the standard value of crop load defined in GB/T51183-2016, for solanaceous and melons, the corresponding value of crop load C_k_ is 0.15 kN/m^2^.

### Concentrated roof load construction E_k_

Concentrated roof loads, usually 0.8 kN m^−2^, are included in the uniformly distributed roof loads.

### Seismic load A_k_

Seismic load due to earthquakes is not considered in the present analysis.

### Snow load S_k_

**Figure 5:**
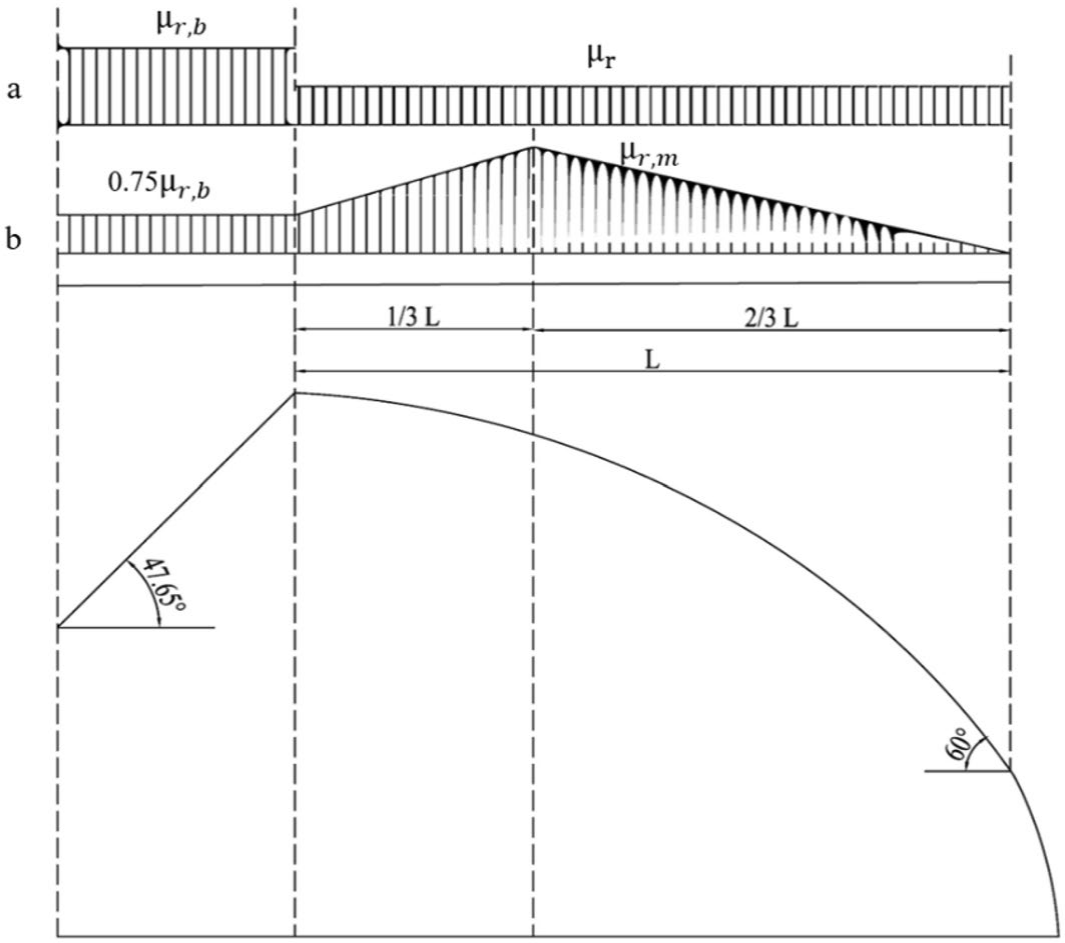
Snow distribution coefficient on the solar greenhouse roof: (**a**) uniform distribution; (**b**) uneven distribution.

Snow load refers to the load acting on the horizontal projection of the solar greenhouse roof. Basic snow pressure is calculated using data of snow weight on open and flat ground. It can be obtained by querying the local snow load table. According to the GB/T51183-2016 parameters, the snow load standard value S_k_ on the horizontal projection of the roof should be calculated with Equation:

10 $${\text{S}}_{{\text{k}}} = \upmu_{{\text{r}}} \cdot{\text{c}}_{{\text{t}}} \cdot{\text{S}}_{0}$$

where S_0_ is the basic snow pressure (kN m^−2^); μ_r_ is the distribution coefficient of roof snow cover; c_t_ is the heating influence coefficient. Considering the effect of snow movement on the greenhouse framework, the distribution coefficient of snow load on the solar greenhouse was divided into uniform distribution and non-uniform distribution (Fig. 5). In the uniform distribution section, μ_r_ was calculated considering the following values: greenhouse span (10 m) and ridge height (6.1 m), μ_r_ = 10/(8 * 6.1) = 0.205; μ_rb_ was related to the elevation angle of the rear roof (47.65°); μ_rb_ = 0.8 * (60°–47.65°)/30° = 0.33. In addition, in the non-uniform distribution section 0.75μ_rb_ = 0.2475. In this case, μ_rm_ was calculated using the following data: greenhouse span (10 m) and ridge height (6.1 m), μ_rm_ = 0.2 + 10 (6.1/10) = 6.3, with a maximum μ_rm_ value of 2.0 when the insulation was covered. According to the GB/T51183-2016 standard, no heating device was included in the solar greenhouse; the heat was stored and release through the wall. For this reason, the c_t_ heating influence coefficient was included in Eq. (1). According to the GB/T51183-2016 standard, the basic snow load is S_0_ = 0.38 kN m^−2^ in the Shenyang, Liaoning Province.

### Governing equations

Greenhouse load conditions are calculated using equation:

11 $${\text{F}} = {\text{P}}\cdot{\text{S}}$$

where F is the force acting on the skeleton (N); P is the force acting on the skeleton per unit area (N/m^2^), which could be constant load (G), crop load (C_k_), concentrated load of roof construction (E_k_) and/or snow load (S_k_); S is the area applied to the skeleton (m^2^).

Equation is used to calculate reference material deformation:

12 $$\uplambda = {\text{F}}/{\text{C}}$$

where λ is the shape variable of the material (mm); F represents the force applied to the material (N). C is the ability of the material to resist deformation, which is known as material stiffness (N mm^−1^).

### Linear buckling stability analysis

Linear buckling analysis is based on the assumption of small displacement theory, the change of structural shape is ignored during the process of loading, and the small incremental displacement is the linear function of external incremental load. In this section, linear buckling analysis was an ideal case, which had some deviations with the actual project. The main purpose of linear buckling analysis was to obtain the upper limit of the actual critical load and buckling mode, which could provide reference for nonlinear stability analysis. Standard for design of steel structures (2017) suggests that the initial defect value of round tube of long-span steel structure should be lower than 1/400, that is 25 mm (10,000 mm/400) for this greenhouse. The initial defect value of square tube of long-span steel structure should be lower than 1/300, that is 33 mm (10,000 mm/300) for this greenhouse. The initial defect value of elliptic tube and Ω tube of long-span steel structure should be lower than 1/350, that is 28.6 mm (10,000 mm/350) for this greenhouse.

### Working conditions

Because the greenhouse is usually subjected to a variety of loads such as snow load and the weight of the rear roof covering, the load distribution on the greenhouse skeleton is relatively complex^21^. For this reason, in the present experiments, constant load + crop load + snow load working conditions were considered. In the cases of uniform (a) and non-uniform (b) distributions snow load were considered for the analysis of four pipe types of greenhouse skeletons. The load combination is shown in Table 4. Also, in the case of non-uniform snow load, the greenhouse skeleton was analyzed considering different beam sizes. In addition, the wall thickness of the main beam was used to determine the safety of the greenhouse skeleton.

**Table 4:** The greenhouse skeleton load combination mode.

Load combinations	Constant load G_k_	Snow load S_k_ (kN/m^2^)	Crop load G_k_ (kN/m^2^)	Concentrated roof load construction E_K_ (kN/m^2^)
a	1.0	0.38u_r,b_, 0.38u_r_	0.15	0.8
b	1.0	0.285u_r,b_, 0.38u_r,m_ ≤ S_k_ ≥ 0.285u_r,b_	0.15	0.8

## Results

Stress and shape are considered as the two main criteria for structural analysis We present the results of the effect of stress and structural deformation on structural performance.

### Effect of uniform snow load on skeleton safety

**Figure 6:**
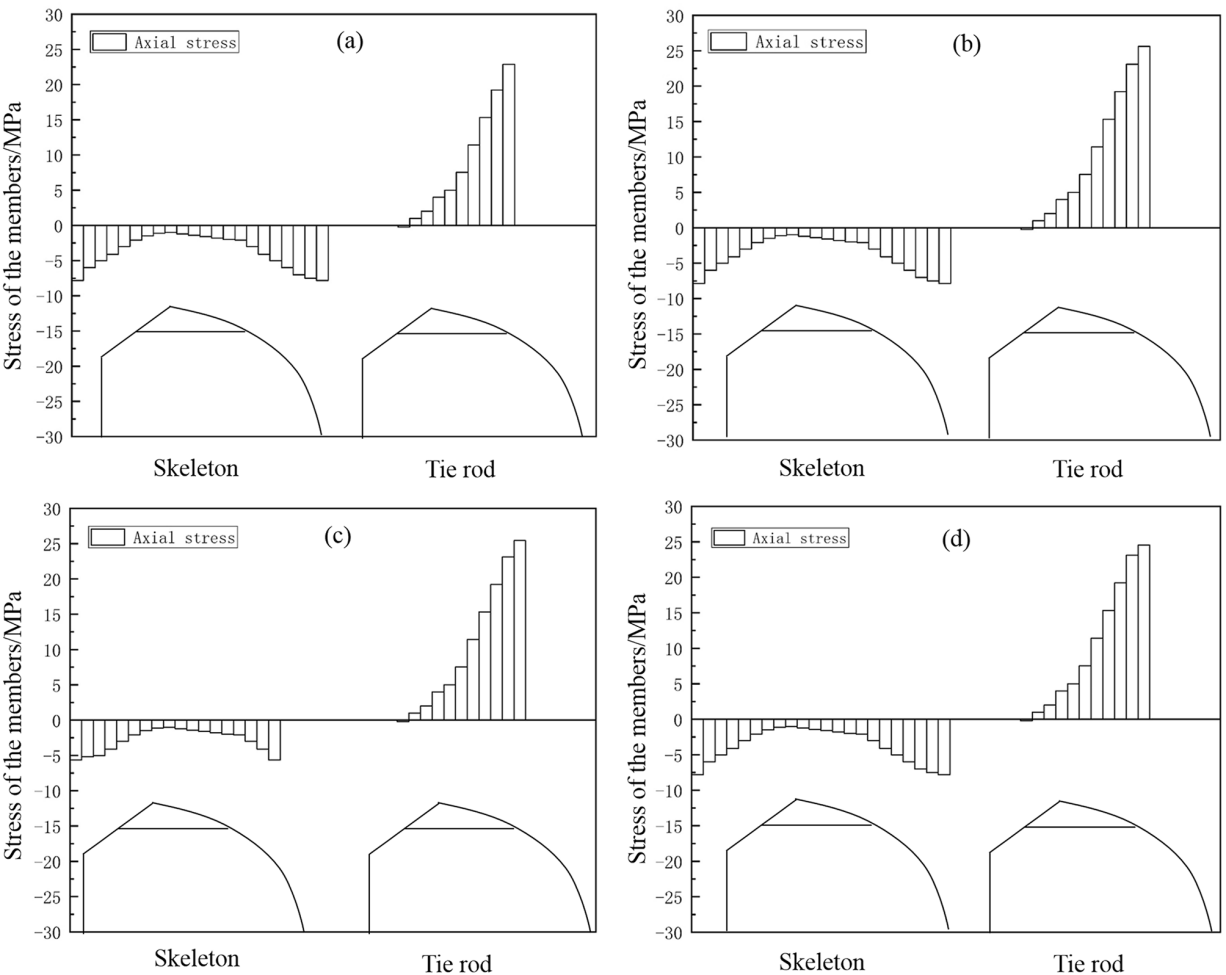
Normal skeleton stress diagram under uniform snow load condition: (**a**) round tube; (**b**) square tube; (**c**) Ω tube; (**d**) elliptic tube.

**Figure 7:**
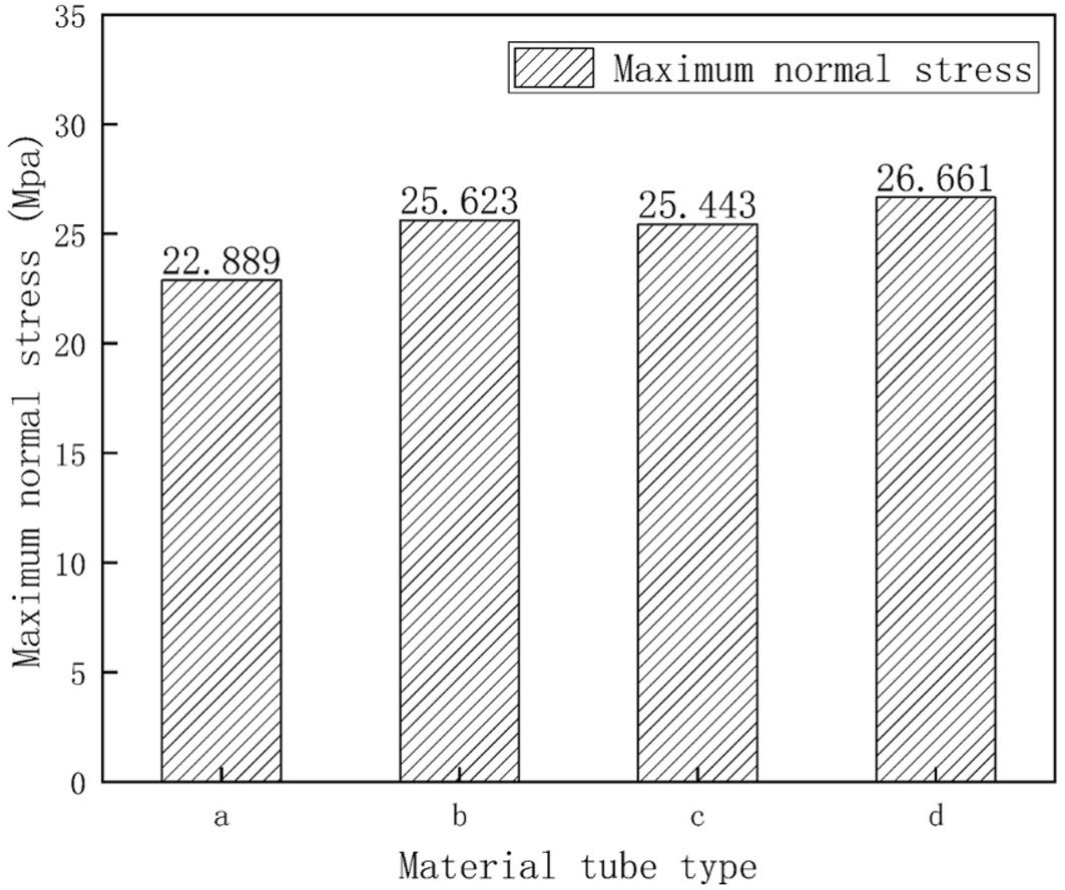
Histogram of normal skeleton stress under uniform snow load condition.

Figure 6 displays the normal stress distribution diagram of the greenhouse skeleton. Results indicated that, under uniform snow load, the maximum normal stress of the skeleton was located at the connection between the transverse tensile reinforcement and the front roof. According to Fig. 7, the maximum normal stress on the four skeleton pipe types were 22.889 MPa, 25.623 MPa, 25.443 MPa and 26.611 MPa, respectively. We concluded that the structural strength of the analyzed pipes complied with the standard requirements. According to the results shown in Fig. 6, the skeleton part with the maximum normal stress was located on the transversal tensile reinforcement, which was subjected to the highest positive pressure. This pressure was the result of the forces exerted by the back slope and the front arch. Data also indicated that under uniform load conditions, the transverse tensile reinforcement was essential in the construction of the greenhouse. Figure 7 shows the comparison for maximum normal stress in the four types of skeleton tubes. According to these data, the round tube displayed the minimum normal stress and elliptic tube the maximum one.

### Greenhouse skeleton shape variations under uniform snow load

**Figure 8:**
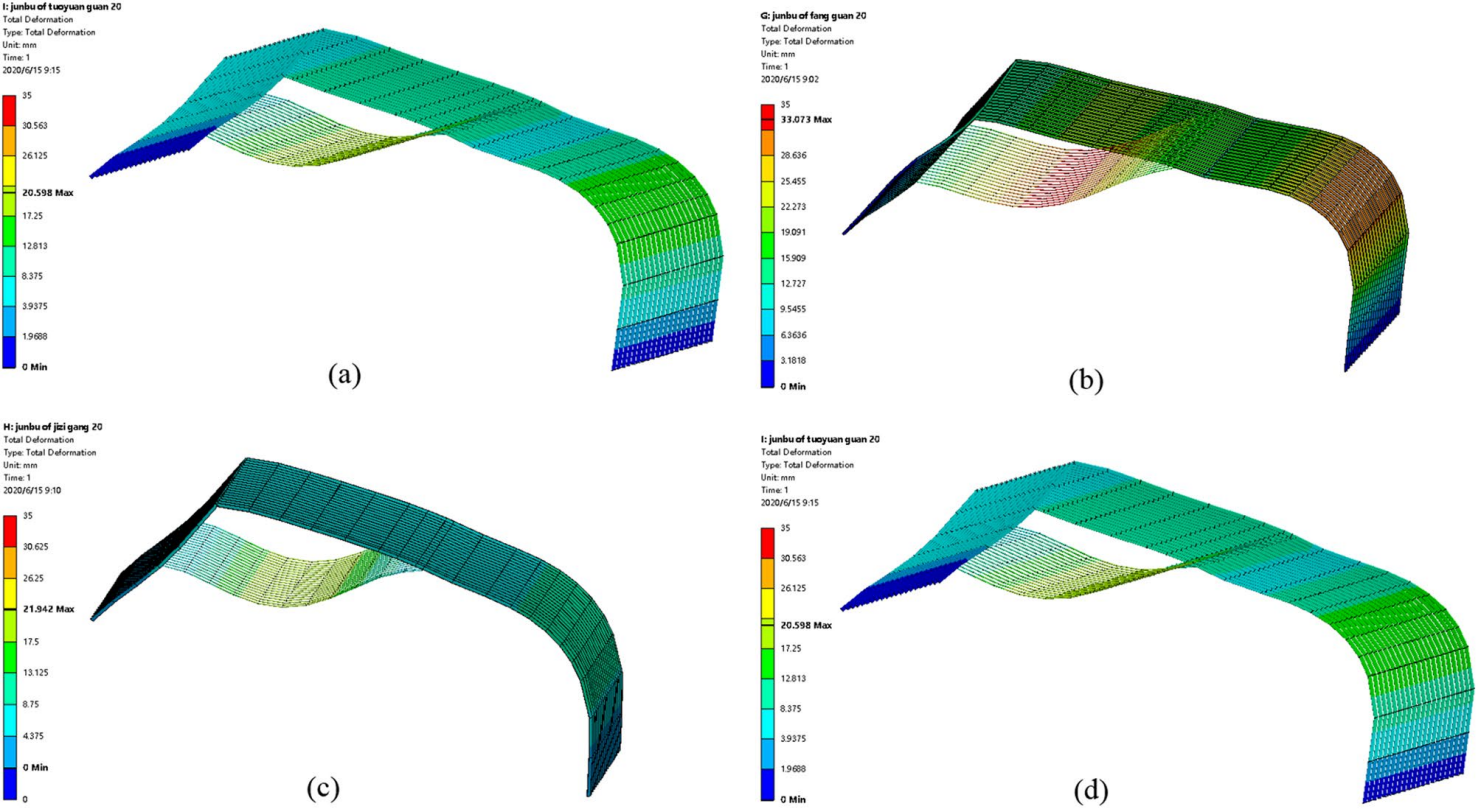
Diagram of skeleton shape variations under uniform snow load condition: (**a**) round tube; (**b**) square tube; (**c**) Ω tube; (**d**) elliptic tube.

**Figure 9:**
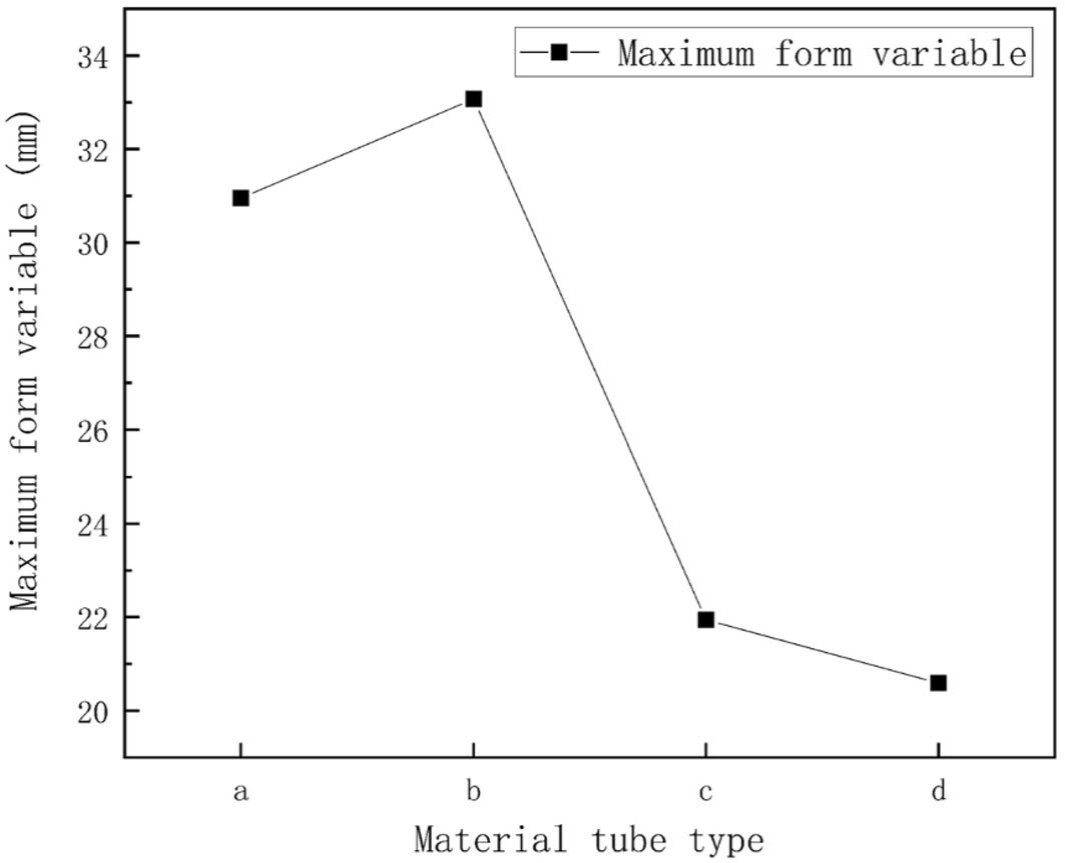
Linear graph for skeleton shape variation under uniform snow load condition: (**a**) round tube; (**b**) square tube; (**c**) Ω tube; (**d**) elliptic tube.

Figure 8 shows the distribution diagram for shape variations of greenhouse skeletons. Data indicated that, under uniform snow load action, the largest shape variations of the greenhouse skeleton were concentrated on the transversal tensile reinforcement. These results were consistent with those obtained for the normal stress distribution of the greenhouse skeleton. The largest shape variations in the four pipe types were 30.952 mm, 33.073 mm, 21.942 mm and 20.598 mm. As Fig. 9 shows, the highest shape variation was that of the square tube. That of the elliptic tube was the smallest one. Therefore, under uniform snow load conditions, the smallest effect on the greenhouse skeleton was the elliptic tube.

### Influence of non-uniform snow load conditions.

**Figure 10:**
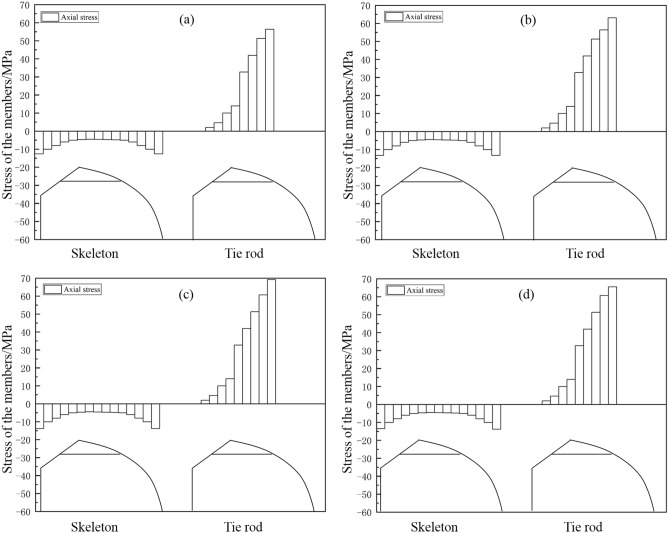
Normal stress diagram of greenhouse skeleton under non-uniform snow load condition: (**a**) round tube; (**b**) square tube; (**c**) Ω tube; (**d**) elliptic tube.

**Figure 11:**
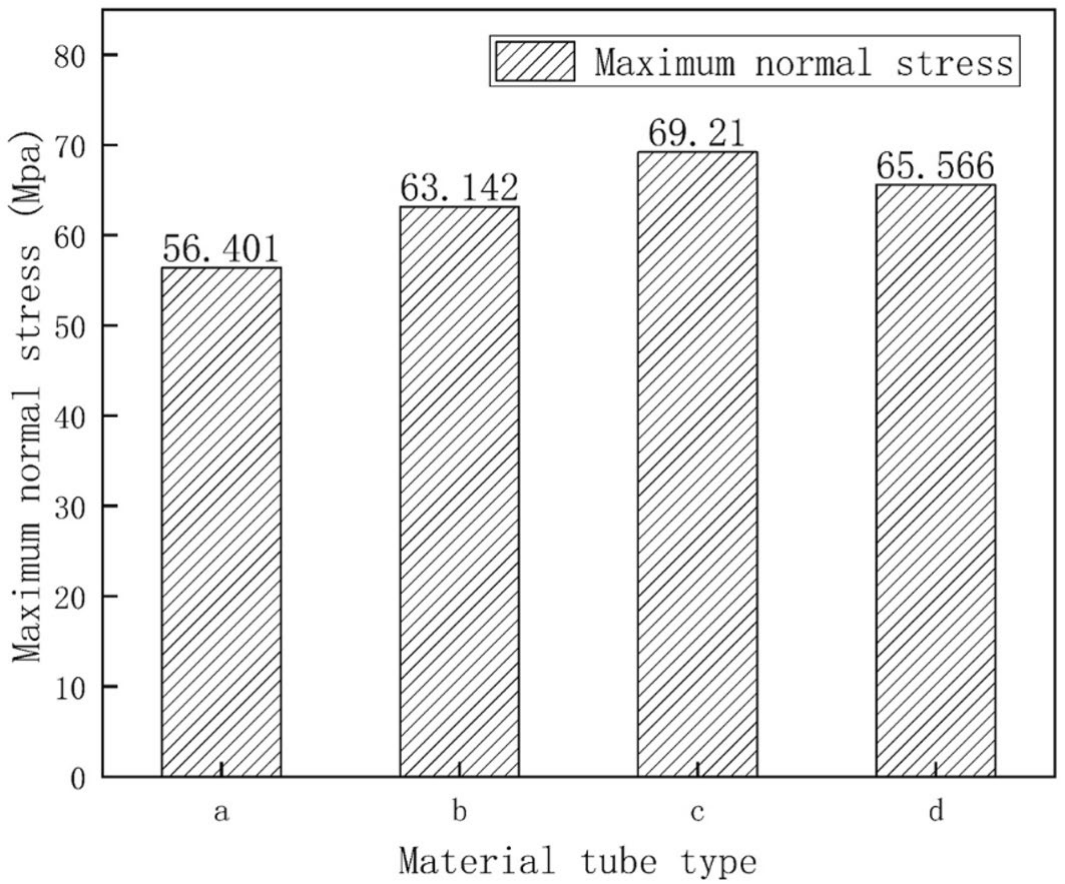
Normal skeleton stress under uneven snow load condition: (**a**) round tube; (**b**) square tube; (**c**) Ω tube; (**d**) elliptic tube.

Figure 10 presents the results for the normal stress distribution of the greenhouse skeleton. Data indicated that under non-uniform snow load conditions, the maximum normal stress of the skeleton was located at the connection between the transversal tensile reinforcement and the front roof. According to Fig. 11, the maximum normal stress on the four skeleton pipe shapes was 56.401 MPa, 63.142 MPa, 69.21 MPa and 65.566 MPa. Comparing these values with that of the Q235 steel, which was 235 MPa, we concluded that these values met the requirements. Also, comparing the results shown in Figs. 6 and 10, we concluded that the skeleton member with the maximum normal stress was the transversal tensile reinforcement. In addition, according to the data presented in Figs. 7 and 11, the normal stress of the skeleton under non-uniform snow load significantly increased. Moreover, comparing the normal stress of the four materials, results indicated that the normal stress corresponding to round tube was the lowest one, and that of the Ω tube was the highest one.

### Greenhouse skeleton shape variations under non-uniform snow load conditions

**Figure 12:**
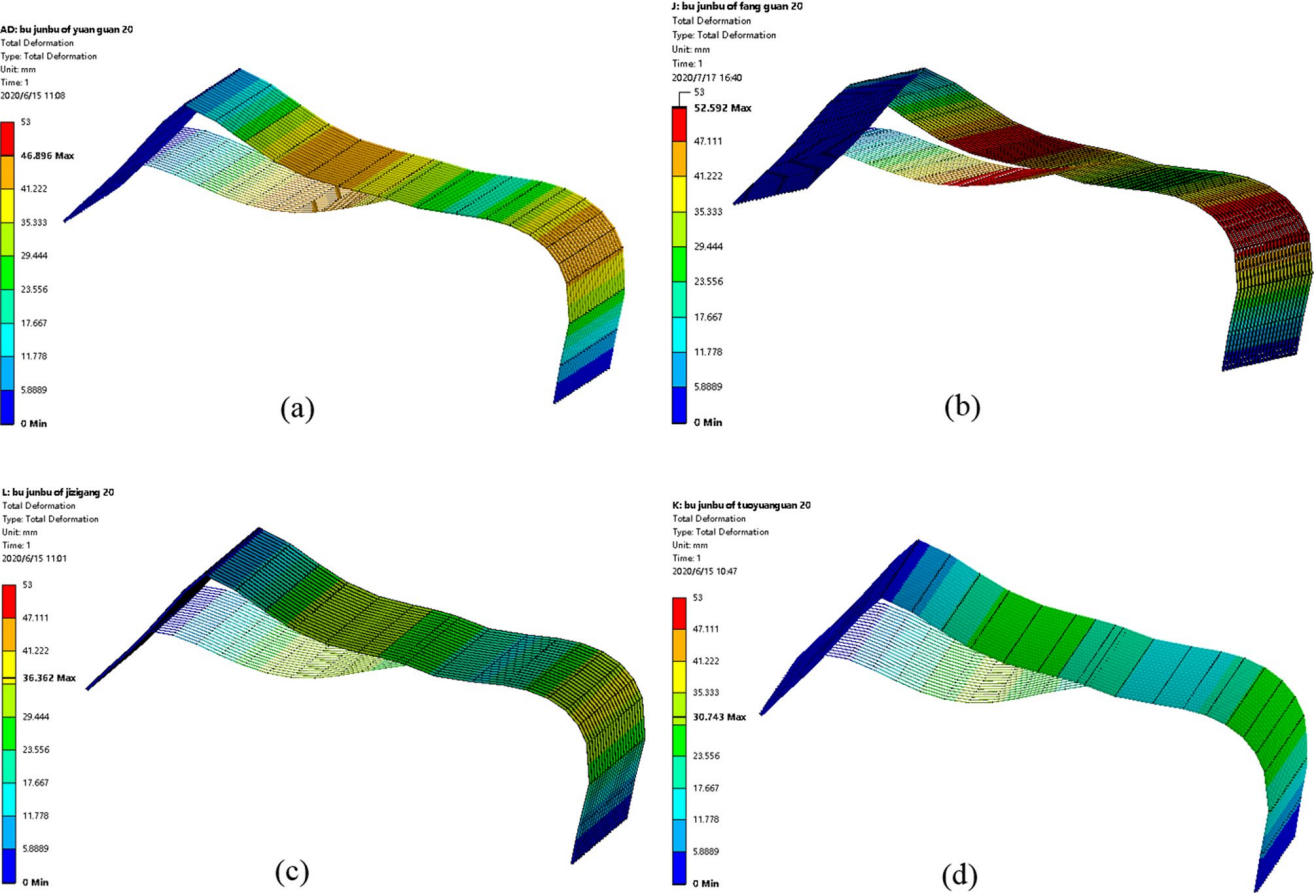
Skeleton shape variations under non-uniform snow load condition: (**a**) round tube; (**b**) square tube; (**c**) Ω tube; (**d**) elliptic tube.

**Figure 13:**
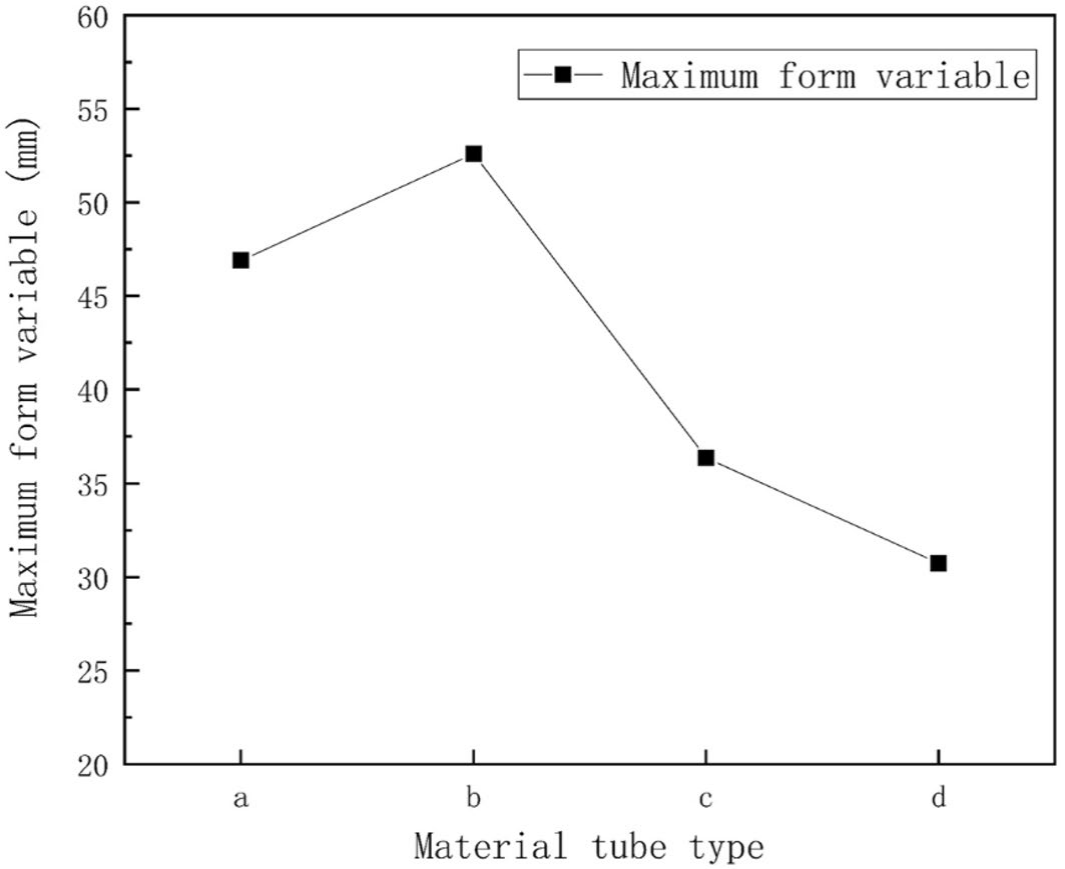
Linear diagram of skeleton variations under non-uniform snow load condition. (**a**) Round tube, (**b**) square tube, (**c**) Ω tube, (**d**) elliptic tube.

Figure 12 displays the distribution of greenhouse skeleton shape variations. Results indicated that under non-uniform snow load conditions, the largest shape variations of the greenhouse skeleton were concentrated on the transversal tensile reinforcement. These results were consistent with those obtained for the normal stress distribution of the greenhouse skeleton. The maximum shape variations of the four-pipe types of greenhouse skeletons were 46.896 mm, 52.592 mm, 36.362 mm and 30.743 mm, respectively. Results presented in Fig. 13 indicated that the square tube skeleton presented the largest shape variation. The elliptic tube greenhouse skeleton displayed the smallest shape variation. Therefore, the smallest effect on the greenhouse skeleton was the elliptic tube.

### Influence of section size on skeleton structural safety

Under non-uniform snow condition, elliptic tubes of different sizes were used as main beams for the greenhouse skeletons. The steel quantity and shape variations of the greenhouse skeletons are shown in Table 5, where T represents the reduction of the greenhouse skeleton shape variation for every increment of 10 kg of steel. T was calculated with the following equation:

13 $${\text{T}} = 10 \, \left( {{\text{L}}_{{1}} - {\text{L}}} \right)/ \left( {{\text{t}} - {\text{t}}_{{1}} } \right)$$

**Table 5:** The quantity and shape of steel used for different materials sizes.

Serial number	Main beam size/mm	Amount of steel (t)/kg	Form of a variable (L)/mm	T/(mm/kg)
1	25 × 40 × 2	902.27	47.766	0
2	25 × 45 × 2	948.96	39.585	1.7522
3	25 × 50 × 2	995.64	33.524	1.5253
4	30 × 60 × 2	1162.3	27.079	0.7956
5	30 × 65 × 2	1209	25.82	0.7155
6	30 × 70 × 2	1255.7	24.796	0.6499
7	30 × 75 × 2	1302.4	23.896	0.5965
8	30 × 80 × 2	1349.1	23.169	0.5269
9	30 × 85 × 2	1395.8	22.444	0.5141
10	30 × 90 × 2	1442.5	21.788	0.4809
11	30 × 95 × 2	1489.2	21.136	0.4537
12	30 × 100 × 2	1535.8	20.597	0.4288

where L_1_ is the greenhouse skeleton shape variation of element number 1; L is the greenhouse skeleton shape variation; t is the amount of steel in the greenhouse skeleton; and t_1_ is the amount of steel in the greenhouse skeleton of the element number 1. The steel structure Design Code (2017) has indicated that the maximum deformation of the elliptic tube in large-span steel structures should be 28.6 mm. The Main girder size 25 × 40 × 2 mm, 25 × 45 × 2 mm and 25 × 50 × 2 mm, our results indicated that shape variations were 47.766 mm, 39.585 mm and 33.524 mm, which exceeded normal shape variations. When the greenhouse skeleton shape variation does not exceed the standard value, with the increase of the greenhouse girder size, the greenhouse skeleton shape variation gradually decreases.

### Influence of wall thickness on skeleton safety

Under non-uniform snow load, elliptic tube of the same size and different wall thickness were used as main beams for the greenhouse skeleton. The steel quantity and shape variations of the greenhouse skeleton are shown in Table 6, where T represents the reduction of the shape variation of the greenhouse skeleton for every 10 kg of increment in steel weight. T was calculated according to the formula T = 10 * (L_1_ − L)/(t − t_1_), L_1_ is the greenhouse skeleton shape variation of Number 1; L is the greenhouse skeleton shape variation; t is the amount of steel in the greenhouse skeleton; and t_1_ is the amount of steel in the greenhouse skeleton of number 1. According to the steel structure Design Code (2017), the maximum elliptic tube deformation in large-span steel structure should be 28.6 mm. Thus, values of 30 × 60 × 1 mm and 30 × 60 × 1.5 mm did not comply with the shape variations standard. According to Table 7, with the increase of wall thickness, the reduction of the skeleton shape variation gradually decreased.

**Table 6:** Amount and shape of steel used for different wall thicknesses.

Serial number	Main girder wall thickness/mm	Amount of steel (t)/kg	Form of a variable (L)/mm	T/(mm/kg)
1	30 × 60 × 1	684.23	35.913	0
2	30 × 60 × 1.5	925.12	29.635	0.2606
3	30 × 60 × 2	1162.3	27.079	0.1848
4	30 × 60 × 2.5	1395.9	25.787	0.1423
5	30 × 60 × 3	1625	24.834	0.1177

**Table 7:** Skeleton size suggestion.

Span/m	7	8	9	10
Main beam size/mm	25 × 45 × 1.5	30 × 60 × 2	30 × 65 × 2	30 × 75 × 2

In the present research, the optimum size of the single tube for the Chinese solar greenhouse skeleton to be used in the Shenyang region was calculated (Table 3). According to our results, the tie rod should be made of a 30 × 2 mm round tube, while the tie bar reinforcement of a 20 × 2 mm round tube.

## Conclusion

In the present research, the effect of different geometric parameters of single tube sections on the safety of solar greenhouse skeletons was studied. The results are provided below:

1. The maximum stress of the four types of cross-section skeletons was located at the connection between the transversal tensile reinforcement and the front roof. This position should be strengthened during construction.
2. Elliptic tube should be selected as the skeleton cross-section during greenhouse construction because it works best in greenhouse loads. It is conducive to the unification of the skeleton standard of the single tube greenhouse.
3. The effect of increasing the greenhouse skeleton size was better than that of increasing the wall thickness of the greenhouse materials.
4. We determined the appropriate skeleton size of single tube skeletons to be used in solar greenhouses for the Shenyang region.
5. The single tube greenhouse skeleton applied to the largest span needs to be carried out in-depth research on it, so as to the main and secondary beam structure of the greenhouse skeleton safety.
